# Preparation and Properties of Plant-Oil-Based Epoxy Acrylate-Like Resins for UV-Curable Coatings

**DOI:** 10.3390/polym12092165

**Published:** 2020-09-22

**Authors:** Jijun Tang, Jinshuai Zhang, Jianyu Lu, Jia Huang, Fei Zhang, Yun Hu, Chengguo Liu, Rongrong An, Hongcheng Miao, Yuanyuan Chen, Tian Huang, Yonghong Zhou

**Affiliations:** 1 College of Materials Science and Engineering, Jiangsu University of Science and Technology, Zhenjiang 212100, China; tangjijunnju@126.com (J.T.); oqqooo12@163.com (J.L.); 2 Institute of Chemical Industry of Forest Products, Chinese Academy of Forestry, Nanjing 210042, China; zdslhs@126.com (J.Z.); hj18252588192@163.com (J.H.); zhangfei@jspi.cn (F.Z.); huyun@icifp.cn (Y.H.); mhc460826041@163.com (H.M.); yuanyuanchen19@163.com (Y.C.); 1551kinoko@gmail.com (T.H.); 3 College of Geographic and Biologic Information, Nanjing University of Posts and Telecommunications, Nanjing 210023, China; 4 College of Chemical Engineering, Nanjing Forestry University, Nanjing 210037, China

**Keywords:** soybean oil, rubber seed oil, wilsoniana seed oil, epoxy acrylate, UV-curable coatings

## Abstract

Novel oil-based epoxy acrylate (EA)-like prepolymers were synthesized via the ring-opening reaction of epoxidized plant oils with a new unsaturated carboxyl acid precursor (MAAMA) synthesized by reacting maleic anhydride (MA) with methallyl alcohol (MAA). Since the employed epoxidized oils including epoxidized soybean oil (ESO), epoxidized rubber seed oil (ERSO), and epoxidized wilsoniana seed oil (EWSO) possessed epoxy values of 7.34–4.38%, the obtained epoxy acrylate (EA)-like prepolymers (MMESO, MMERSO, and MMEWSO) indicated a C=C functionality of 7.81–4.40 per triglyceride. Furthermore, effects of the C=C functionality and the addition of hydroxyethyl methacrylate (HEMA) diluent on the ultimate properties of the resulting UV-cured EA-like materials were investigated and compared with those of commercially available acrylated ESO (AESO) resins. As the C=C functionality increased, the storage modulus at 25 °C (*E’*_25_), glass transition temperature (*T*_g_), 5% weight–loss temperature (*T*_5_), tensile strength and modulus (*σ* and *E*), and hardness of the coating for both the pure EA and EA/HEMA resins increased significantly as well. These properties indicated similar trends when comparing the EA materials with 30% of HEMA with those pure EA materials. Specially, although ERSO had a clearly lower epoxy value that ESO, both the UV-cured pure MMERSO and MMERSO/HEMA materials showed much better *E’*_25_, *T*_g_, *σ*, and *E* than their AESO counterparts, indicating that the MAAMA modification of epoxidized plant oils was much more effective than the modification of acrylic acid to achieve high-performance oil-based epoxy acrylate resins.

## 1. Introduction

The ultraviolet (UV)-curing technique has received enormous interest in modern industrial areas such as coatings, inks, and adhesives due to its distinct advantages, including being efficient, energy-saving, enabling, economical, eco-friendly, etc. [1,2,3,4,5,6]. The global market of UV-curable resins was nearly $3.1 billion in 2015 and is expected to reach $4.6 billion in 2020, with an annual growth rate close to 8.7% for the period 2015–2020 [7]. However, most of the raw materials for UV-curable coatings currently available in the global market are from non-renewable, environmentally polluting petroleum-based fossil resources. Therefore, developing UV-curable coatings by introducing biorenewable components such as carbohydrates, plant oils, and rosins related to sustainability and environmental protection is required to match sustainable development strategies [8,9].

Plant oils have the features of environmentally benign, abundance, biodegradability, and triglyceride structures; thus, they have been used as an ideal substitute to prepare bio-based prepolymers for UV-curable coatings [10,11,12,13,14,15,16,17,18,19]. Plant-oil-based epoxy acrylate (EA) is one of the common UV-curable prepolymers, which is usually prepared through the ring-opening reaction of epoxidized plant oils with acrylic acid (AA) or its derivatives. Acrylated epoxidized soybean oil (AESO), perhaps the most often bio-based UV-curable prepolymer, has been proverbially employed in the fields of coatings and adhesives [7,15,20,21,22]. However, most of the reported oil-based EA resins usually possessed low stiffness and heat resistance, which greatly limited their application in the areas where petroleum-based EA resins can be used. For example, the tensile modulus and *T*_g_ of bisphenol A epoxy acrylate resin reported by Xiao et al. could reach 300–500 MPa and 75 °C, respectively, while the pure AESO resin only showed a tensile modulus of around 60 MPa and *T*_g_ of about 20 °C due to its lack of rigid structure [23,24]. Therefore, a variety of works dedicated to improving such properties for oil-based EA resins have been conducted [7,20,21,22,25,26,27,28,29], among which the design of new oil-based EA prepolymers has gained much attention. For instance, Li et al. [22] synthesized a novel EA prepolymer from soybean oil (SO) through melt ring-opening reaction of epoxidized soybean oil (ESO) with monomethyl itaconate. However, the resulting monomethyl itaconated epoxidized soybean oil (IESO) materials generally exhibited no clear improvement in strength, stiffness, and heat resistance in comparison with the AESO counterparts, which is probably attributed to that the C=C functionality of IESO (2.39 per ESO) being similar to that of AESO (2.25 per ESO). In our group, we also developed a novel SO-based EA prepolymer through the modification of ESO with an unsaturated carboxylic acid precursor (HEMAMA) prepared by reacting hydroxyethyl methacrylate (HEMA) with maleic anhydride (MA) [30]. Due to the precursor having two active C=C groups and a side methyl group, the resultant SO-based EA prepolymers possessed both high functionality (5.51–6.05 per ESO) and the structure of steric hindrance. Therefore, the properties such as tensile strength and modulus, storage modulus at 25 °C, *T*_g_, and pencil hardness for the UV-cured EA materials were all greatly improved compared with the AESO material. However, up until now, novel oil-based EA resins with high performance are still very scarce, and whether the important properties can be tuned by varying the renewable raw materials such as epoxidized plant oils are unknown.

Soybean oil, with a global production that amounted to 56.52 million tons in 2019/2020, has become a very attractive bio-based alternative to petroleum-based compounds in polymeric materials [10]. In addition, some woody plant oils such as rubber seed oil (RSO) and *wilsoniana* seed oil (WSO) also make up a great proportion of the current consumption of bio-based feedstocks in the chemical industries as they do not compete with agricultural food production [31,32,33]. In 2013, the global total production of natural rubber latex was estimated to reach 11.9 million tons, and this figure is expected to increase significantly with the further expansion of planting areas [34]. *Wilsoniana* tree is also a widely distributed woody oil plant in the south of China, and the annual production of WSO could reach 30 million tons [33]. The RSO and WSO have been used in the areas of coatings [35,36,37], plasticizers [38], and some other fields [33,39]. Nevertheless, up to now, their employment in the construction of UV-curable coatings has not been reported yet.

In this paper, we aim to develop high-performance oil-based EA resins using epoxidized plant oils with different epoxy values. According to the above analysis, incorporating high functionality and the structure of steric hindrance into the new EA prepolymers is an effective way to fulfill the purpose. Therefore, a new unsaturated carboxyl acid precursor (MAAMA), obtained through the reaction of methallyl alcohol (MAA) with maleic anhydride (MA), was used to modify epoxidized plant oils including ESO, epoxidized RSO (ERSO), and epoxidized WSO (EWSO). It should be noted that the employed epoxidized plant oils possessed a decreasing epoxy value from 7.34% to 4.38%, thus resulting in the corresponding EA-like prepolymers with an introduced C=C functionality of 7.81 to 4.40 per triglyceride. In addition, the effects of C=C functionality and incorporating HEMA diluent on the physiochemical properties and UV-curing behaviors of the resultant oil-based EA resins were investigated and compared with commercial AESO resins.

## 2. Experimental

### 2.1. Materials

ESO was obtained from Shanghai Aladdin Chemistry Co., Ltd. (Shanghai, China), and possessed an epoxy value of 7.34%. RSO and WSO were provided by Southwest Forestry University (Yunnan, China). AESO with a viscosity of 37,500 mPa s, was supplied by Shandong Shouguang Luke Chemical Co., Ltd. (Shandong, China). The methallyl alcohol (MAA, 98%) and hydroxyethyl methacrylate (HEMA, ≥97%) were provided by Macklin Chemical Reagent Co., Ltd. (Shanghai, China). Maleic anhydride (MA, ≥99.5%) was provided by Nanjing Chemical Co., Ltd. (Nanjing, China). Triphenylphosphine (TPP, ≥98%) was supplied by Sinopharm Chemical Reagent Co., Ltd. (Shanghai, China). 4-Methoxyphenol (MEHQ, ≥99%) was supplied by Shanghai Titan Technology Co., Ltd. (Shanghai, China). Darocur 1173 (98%) was provided by Saen Chemical Technology Co., Ltd. (Zhengzhou, China).

### 2.2. Synthesis of MAAMA

**Scheme 1:**
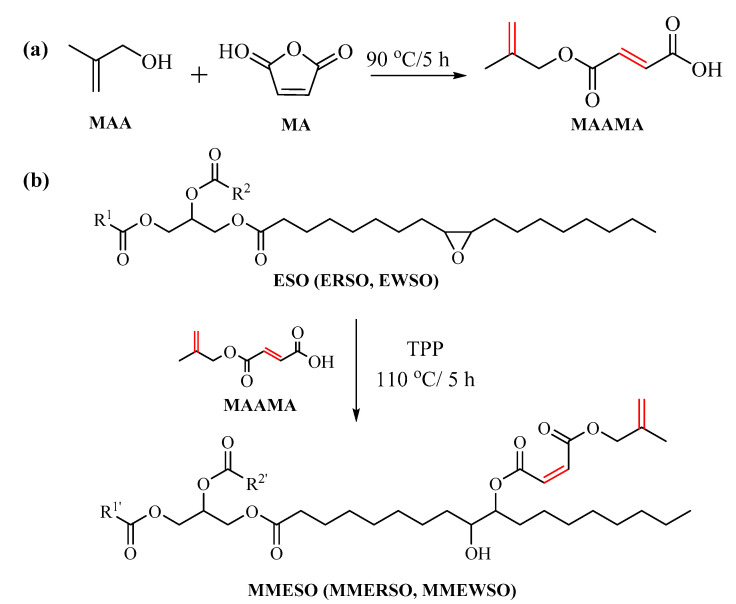
Synthesis route for new unsaturated carboxyl acid precursors obtained through the reaction of (**a**) methallyl alcohol (MAA) with maleic anhydride (MAA) (MAAMA) and (**b**) obtained epoxy acrylate-like prepolymers MMESO, MMERSO, and MMEWSO. ESO: epoxidized soybean oil, ERSO: epoxidized rubber seed oil, EWSO: epoxidized wilsoniana seed oil.

About 28.5 g of MAA, 39.2 g of MA, and 0.136 g of MEHQ were mixed into a 250 mL four-neck round-bottom flask fitted with a mechanical stirrer, thermometer, refluxing condenser, and a nitrogen inlet. Then, the reaction was performed at 70 °C under the protection of N_2_ until solidified MA completely melted. Subsequently, the mixture was heated to 90 °C for 5 h, and a golden yellow transparent liquid at room temperature was obtained (see Scheme 1 for synthesis route).

### 2.3. Epoxidation of Plant Oils

ERSO was synthesized as follows: approximately 42.7 g of RSO, 4.5 g of formic acid, and 50 mL of toluene were mixed in a four-necked round-bottom flask. Subsequently, about 3.0 g of concentrated sulfuric acid and 51 g of 30% hydrogen peroxide were added dropwise into the flask under 50 °C within 30 min. After that, the mixture was heated to 70 °C for 5 h. Finally, the product was washed by distilled water until neutral, and then a pale yellow transparent liquid that possessed an epoxy value of 5.15% was obtained. EWSO was synthesized in the same way, and its epoxy value was 4.38%.

### 2.4. Synthesis of MMESO, MMERSO, and MMEWSO

The synthesis methods of MMESO, MMERSO, and MMEWSO are similar, taking the synthesis of MMESO as an example (Scheme 1). Typically, 50 g of ESO, 35.83 g of MAAMA, 0.086 g of MEHQ, and 0.86 g of TPP were charged into a four-neck round-bottom flask. Under the protection of N_2_, the mixture was agitated under 110 °C for 2 h. During the work-up procedures, the resulting crude MMESO product was washed by 10 wt % NaCl/H_2_O solutions three times, dissolved by dichloromethane, dried with MgSO_4_, and evaporated via rotary evaporation. Finally, a light-yellow, viscous liquid product at room temperature was obtained.

### 2.5. Curing of MMESO, MMERSO, and MMEWSO Resins

The UV-curable resins were obtained by mixing pure EA-like prepolymers (MMESO, MMERSO, or MMEWSO), HEMA, and Darocur 1173 photoinitiator at room temperature. The formulations of EA or EA/HEMA resins are shown in Table 1. Take the synthesis of MMESO/HEMA30 as an example: about 7.0 g of MMESO, 3 g of HEMA, and 0.15 g of Darocur 1173 were mixed in a round-bottom flask fitted with a mechanical stirrer. Then, the mixture was agitated at room temperature for about 30 min, followed by degassing under vacuum for about 10 min. Then, the resulting samples were cast into a self-made polytetrafluoroethylene mold or coated on polished tinplate sheets by a filmmaker. Finally, the casted or coated samples were cured by an Intelli-Ray 400W UV light-curing microprocessor with a UV wavelength of 320–390 nm from Uvitron International Corporation (USA). The exposure intensity and time for all the samples were 100 mW/cm^2^ and 20 min, respectively.

### 2.6. Characterization

#### 2.6.1. Acid Value (*Av*)

Acid values of samples were determined based on the procedures outlined in GB/T 2895-1982, as provided in a previous work of us [40].

#### 2.6.2. Epoxy Values (*E*)

Epoxy values were recorded based on the procedures outlined in GB 1677-81. Approximately 0.5 g of the sample was completely dissolved in 20 mL of chlorhydric acid/acetone solution; then, bromocresol was added as an indicator. Finally, the solution was titrated by 0.15 mol/L NaOH solution. The epoxy value (g/100g) of the sample was determined using the following equation:

(1) $$E=\frac{(V_{1}-V_{2})\times C_{\mathrm{NaOH}}\times 16}{10m}$$

where *V*_1_ = blank burette reading (mL), *V*_2_ = sample burette reading (mL), *C*_NaOH_ = initial concentration of NaOH solution, 16 = molar mass of oxygen atom, *m* = weight of sample (g).

#### 2.6.3. Fourier Transform Infrared Spectroscopy Analysis (*FT-IR*)

FT-IR tests were conducted on a Nicolet iS10 IR spectrometer from Thermo-Fisher Corporation (Thermo-Fisher Corporation, Waltham, Massachusetts, USA) within a scanning range from 650 to 4000 cm^−1^.

#### 2.6.4. Nuclear Magnetic Resonance Analysis (*NMR*)

^1^H NMR tests were conducted on a DRX-300 Advance NMR spectrometer from Bruker Corporation (Bruker Corporation, karlsruhe, Germany) with CDCl_3_ as a solvent.

#### 2.6.5. Gel Content (*Cgel*)

The *C*_gel_ tests of MMEVO samples were performed via Soxhlet extraction. Typically, approximately 0.5 g of the cured samples were precisely weighed (recorded as *m*_0_), extracted by acetone for 24 h, and finally dried at 60 °C until a constant mass was obtained (recorded as *m*_1_). The *C*_gel_ values of samples were calculated as *m*_1_/*m*_0_ [41].

#### 2.6.6. Dynamic Mechanical Analysis (*DMA*)

The DMA test of the samples was carried out on a Q800 solids analyzer (TA Corporation, New Castle, PA, USA) under the conditions including a stretching mode, a frequency of 1 Hz, a temperature within a range of −50 to 200 °C, and a heating rate of 3 °C/min. The size of the tested samples was about 40 × 6 × 1 mm^3^.

#### 2.6.7. Thermogravimetric Analysis (*TGA*)

The TGA test of the samples was performed using an STA 409PC thermogravimetry instrument from Netzsch Corporation (Netzsch Corporation, bavaria, Germany) under N2 at a flow rate of 100 mL/min. The cured samples were ground to powders before the test. About 10 mg of the powder for each sample was tested in a temperature range of 40 to 600 °C and at a heating rate of 15 °C/min.

#### 2.6.8. Mechanical Properties

The tensile properties of samples were analyzed using a UTM 4304 universal tester (Shenzhen Suns Technology Corporation, Shenzhen, China) with a speed of 5 mm/min. Five specimens with a size of 80 × 10 × 1 mm^3^ were evaluated for each sample to calculate the average values.

#### 2.6.9. Coating Properties

Adhesion of the UV-cured coatings was evaluated by an adhesion test machin (Tianjin Shiboweiye Glass Instrument Corporation, Tianjin, China) according to the procedures specified in GB 1720-79(89). The adhesion grade ranged from 1 to 7 grade (1 grade is the best). The pencil hardness of the UV-cured coatings was measured on a QHQ-A pencil hardness tester from Tianjin Litengda Instrument Corporation based on the procedures listed in GB/T 6739-2006. The pencil hardness mainly includes 6H to H, HB, and B to 6B (from the hardest to the softest). Flexibility of the UV-cured coatings was tested by a QTY-32 paint film cylindrical bending machine from Tianjin Litengda Instrument Corporation (China) according to the procedures listed in GB/T 1731-93. The class of flexibility involves 2, 3, 4, 6, 8, 10, 12, 14 mm, etc., (2 mm is the best). Detailed procedures for the coating tests were indicated in a previous work of ours [30,42].

#### 2.6.10. UV-Curing Kinetics

The UV curing behaviors of the obtained liquid resins were tested by a modified Nicolet 5700 spectrometer (Thermo-Nicolet Instrument Corporation, Madison, WI, USA). The C=C conversion rate (*α*_C = C_) was recorded by monitoring the absorption intensity of the C=C peak at approximately 810 cm^−1^, which can be calculated using the following equation [43,44]

(2) $$\alpha_{C=C}=\frac{A_{0}-A_{t}}{A_{0}}\times 100\%$$

where *A*_0_ and *A*_t_ are the C=C peak areas at the original time and *t* time, respectively.

## 3. Results and Discussion

### 3.1. Synthesis and Characterization of Oil-Based EA-Like Prepolymers

The synthesis of pure EA-like prepolymers mainly involved two steps, as displayed in Scheme 1. *A*_v_ values of the products were used to monitor the reaction extents. First, the synthesis process of the MAAMA precursor was investigated, as shown in Figure S1. After being performed at 90 °C for 5 h, its *A*_v_ value was basically stable at around 329.8 mgKOH/g, which was very close to the theoretical acid value of 332.1 mgKOH/g. Second, MAAMA was used to modify ESO, ERSO, and EWSO, and after being reacted at 90 °C for 2 h, the *A*_v_ values of the reaction mixtures were all below 25 mgKOH/g (as shown in Table 2). Finally, the corresponding EA-like precursors (MMESO, MMERSO, and MMEWSO) products were obtained.

**Figure 1:**
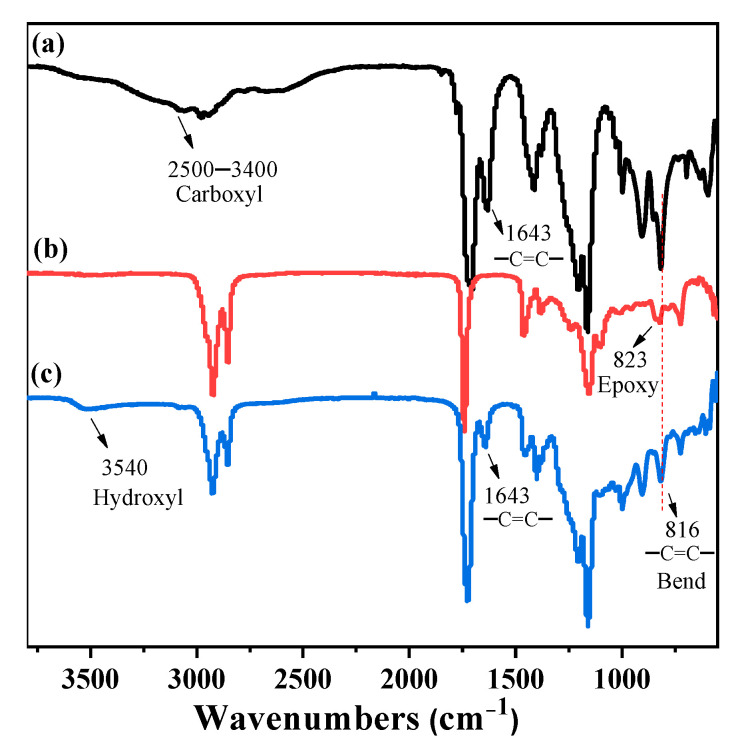
FT-IR spectra of (**a**) MAAMA, (**b**) ESO, and (**c**) MMESO.

Chemical structures of the MAAMA, ESO, and final EA-like prepolymers MMESO were characterized by FT-IR, as depicted in Figure 1. The FT-IR spectra of ERSO, MMERSO, EWSO, and MMEWSO are depicted in Figure S2. In the MAAMA spectrum, the strong absorption bands around 2500–3400 cm^−1^, 1643 cm^−1^, and 816 cm^−1^ were attributed to the characteristic peaks of carboxyl groups, C=C extensional vibration, and C=C bend vibration, respectively [45]. In the spectra of ESO, ERSO, and EWSO, the peaks at 823 cm^−1^ related to epoxy groups were observed [23]. As for the spectra of MMESO, MMERSO, and MMEWSO, the epoxy peak at 823 cm^−1^ and the carboxyl peak at 2500–3400 cm^−1^ basically disappeared, while a new small peak at around 3540 cm^−1^ assigned to the hydroxyl group appeared [22,46,47]. These changes indicated that the epoxy groups on epoxidized plant oils (EPO) successfully reacted with the carboxyl group on MAAMA. Besides, new peaks at 816 and 1643 cm^−1^ appeared, which could be assigned to the C=C bend vibration and C=C stretching vibration, respectively. All these changes indicated that EPO has been successfully grafted by MAAMA.

**Figure 2:**
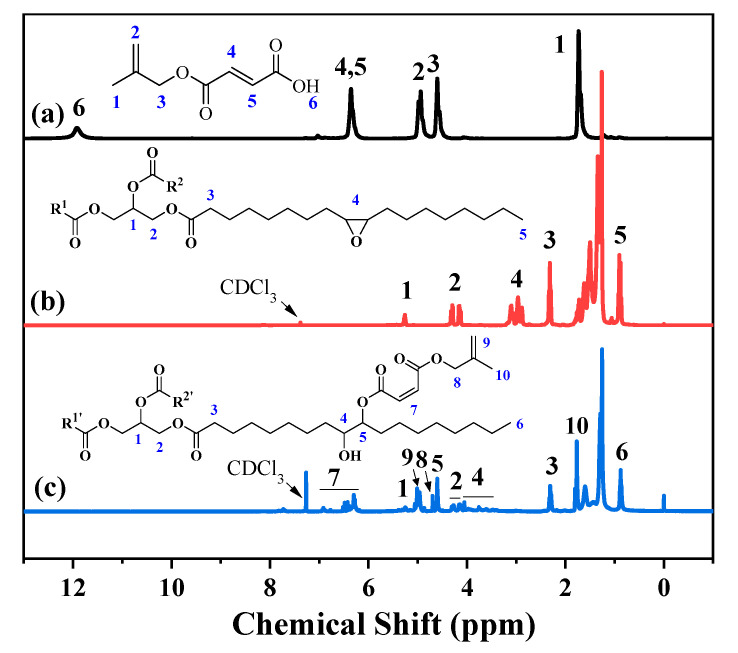
^1^H NMR spectra of (**a**) MAAMA, (**b**) ESO, and (**c**) MMESO.

The ^1^H NMR spectra of MAAMA, ESO, and MMESO are presented in Figure 2. The ^1^H NMR spectra of ERSO, MMERSO, EWSO, and MMEWSO are depicted in Figure S3. In the spectrum of MAAMA, the peak at 11.9 ppm corresponds to the protons of carboxyl groups due to the ring-opening reaction of MA [30]. The peaks at 4.94 and 6.37 ppm could be assigned to the C=C protons on methallyl ester and maleate, respectively [4]. The peaks at 1.73 ppm were assigned to the three-terminal methyl protons in methallyl alcohol, which are always used as a reference since its intensity should not alter during the modification. According to Figure S4, the C=C functionality of MAAMA can be calculated from the peaks at 4.8–5.1 ppm and 6.0–6.9 ppm and it was 1.98, which was very close to the theoretical value of 2. In the spectra of ESO, ERSO, and EWSO (Figure 2 and Figure S3), the peaks at 2.8–3.2 ppm correspond to the protons on epoxy groups [48]. The peaks at around 0.88 ppm were assigned to the terminal methyl protons in triglycerides, which can be used as a reference to estimate the amount of epoxy group, since it is inactive throughout the modification process. Based on Figures S5–S7, the introduced oxirane groups per triglyceride of ESO, ERSO, and EWSO were 4.36, 3.0, and 2.53, respectively. In the spectra of MMESO, MMERSO, and MMEWSO (Figure 2 and Figure S3), the peaks at 11.9 ppm and 2.8–3.2 ppm respectively corresponding to the carboxyl groups and the epoxy group almost disappeared, indicating the reaction between them. Meanwhile, a new peak at about 4.0 ppm attributed to the methine protons on the connecting structure of MAAMA and ESO, ERSO, and EWSO occurred [30]. Besides, new strong peaks at around 4.8–5.1 ppm and 6.0–6.9 ppm appeared, which can be ascribed to the introduced C=C protons from MAAMA. The introduced C=C functionality on the EA-like prepolymers could also be calculated according to the reference peak at 0.88 ppm. Based on Equation (3) and Figures S8–S10, the introduced C=C functionality for ESO, ERSO, and EWSO was 7.81, 5.92, and 4.40, respectively, which was much higher compared to the commercial AESO products (2.25, see Equation (S1) and Figure S11) [40].

(3) $$N_{C=C}=\frac{(A_{4.8–5.1\;\mathrm{ppm}}+A_{6.0–6.9\;\mathrm{ppm}})/2}{A_{0.88\;\mathrm{ppm}}/\;9}=\frac{9(A_{4.8–5.1\;\mathrm{ppm}}+A_{6.0–6.9\;\mathrm{ppm}})}{2A_{0.88\;\mathrm{ppm}}}$$

In theory, the C=C functionality introduced (*N*_C=C_) in MMESO, MMERSO, and MMEWSO should be twice the amount of epoxy groups consumed (*C*_epoxy_) in ESO, ERSO, and EWSO, respectively. In reality, the C=C functionality introduced was slightly less than twice the oxirane group consumption. This may be due to the incomplete reaction or side reactions that occurred during EA synthesis. For instance, it could allow an oligomerization between oxirane and oxirane groups caused by the residual MAA or MA [26,30,49].

### 3.2. Bio-Based Content of the UV-Cured EA-Like Materials

The bio-based content of a material can be defined as the amount of bio-based carbon as a percentage of the total organic carbon weight in the product [2,42,47]. According to this definition, the bio-based contents of the neat ESO, ERSO, EWSO, MAAMA, HEMA, HEA, and Darocur 1173 were 100%, 100%, 100%, 0%, 0%, 0%, and 0%, respectively. The bio-based contents of the resulting materials are listed in Table 2. As the C=C functionality increased, the bio-based content of the UV-cured pure EA resins decreased gradually. Since the pure MMESO material possessed the highest C=C functionality, it presented the lowest bio-based carbon content of 59.0%. In contrast, AESO contained the lowest C=C functionality and therefore exhibited the highest content up to 84.1%. Similar trends were observed for the EA/HEMA materials. In addition, a significant decrease in the bio-based content of EA-like resin was observed when incorporating 30% of HEMA diluent. For instance, the bio-based content of MMESO/HEMA30 dropped from 59.0% in pure MMESO to 41.3%.

### 3.3. Gel Contents of the UV-Cured EA-Like Materials

Gel content (*C*_gel_) is closely related to the final properties of thermosetting materials because it can reflect the cross-link extent of the thermosets [2]. The *C*_gel_ values of the UV-cured EA or EA/HEMA materials are displayed in Table 2. The UV-cured pure MMESO and MMERSO materials demonstrated a higher *C*_gel_ than commercially available AESO resins, suggesting that they had a higher cross-link extent than AESO. Similar trends were observed when comparing the three EA/HEMA30 materials with the AESO/HEMA30 materials. As the C=C functionality on EPO rose from 4.40 to 7.81, the *C*_gel_ values of the pure EA materials increased from 87.1% to 95.9%, and the values of the EA/HEMA materials increased from 98.5% to 99.9%. These results indicated that the improved functionality of thermosets can effectively enhance the cross-link extent of the resulting UV-cured materials. Furthermore, the *C*_gel_ values of the cured EA/HEMA resins containing 30% of HEMA diluent were apparently improved compared to those of pure EA resin, which demonstrated that the incorporation of diluent was beneficial to the improvement of the cross-linking of the UV-cured materials.

### 3.4. Properties of the UV-Cured EA-Like Materials

#### 3.4.1. Dynamic Mechanical Analysis

**Figure 3:**
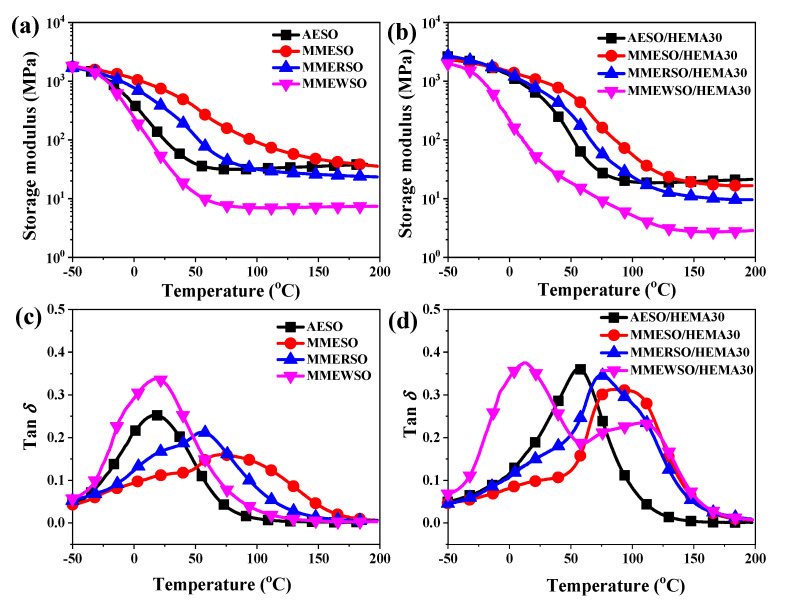
Storage modulus (**a**,**b**) and loss factor(**c**,**d**) of the UV-cured EA-like materials.

The dynamic mechanical analysis including the storage modulus (*E*’) and loss factor (tan *δ*) of the UV-cured EA-like samples is demonstrated in Figure 3, and the related data are summarized in Table 3. The glass transition temperature (*T*_g_) was defined as the peak temperature of tan *δ* curves, and the cross-link density (*ν*_e_) of the cured resins was calculated using the following equation [2,22,50]

(4) $$\nu_{e}=\frac{E^{\prime }}{3RT}$$

where *E*’ is the storage modulus of the resins in the rubber state (the *E*’ at *T*_g_ + 60 °C was selected to calculate *ν*_e_ in this work), *R* represents the gas constant, and *T* represents the absolute temperature. Firstly, the pure MMESO material showed the *E*’ at 25 °C (*E′*_25_) values of 697.5 MPa and *T*_g_ values of 69.4 °C, which were 7.0 and 4.4 times the values of the neat AESO material, respectively. The pure MMERSO material also showed much better *E’*_25_ and *T*_g_ than the pure AESO material, although ERSO had a clearly lower epoxy value that ESO. Similar results were demonstrated when comparing the MMESO/HEMA30 and MMERSO/HEMA30 materials with the AESO/HEMA30 material. All the results indicated that the MAAMA modification of epoxidized plant oils was much more effective to achieve high-performance plant oil-based EA resins than the common acrylic acid (AA) modification. Secondly, for the UV-cured pure EA materials, as the C=C functionality increased, the *E’*_25_ improved from 42.6 to 697.5 MPa, *T*_g_ improved from 17.2 to 69.4 °C, and *ν*_e_ increased from 0.86 × 10^3^ to 5.74 × 10^3^ mol/m^3^. The improvement of *E’*_25_ and *T*_g_ probably results from the increase of *ν*_e_ and the incorporation of more methyl steric structure from MAA. Similar trends were observed for the EA/HEMA materials, except that the MMEWSO/HEMA30 system demonstrated two *T*_g_ values, which indicated the existence of phase separation. For instance, the MMESO/HEMA30 material showed a *T*_g_ value of 84.0 °C, which was clearly higher than that of bisphenol A epoxy acrylate resin [24]. In addition, compared to the pure EA systems, the *E′*_25_ and *T*_g_ values of the EA/HEMA materials increased significantly, while the *ν*_e_ values decreased during this change. The decline of *ν*_e_ values probably lies in that the added diluent is a monofunctional monomer, which increased the effective molecular weight between cross-linked sites [41]. Therefore, the growth of *E′*_25_ and *T*_g_ values can be ascribed to the methyl steric hindrance from HEMA [22,41]. These results also suggested that the steric structure of HEMA played a more crucial part than the *ν*_e_ values in determining the thermal–mechanical properties of the novel EA-like materials.

#### 3.4.2. Thermogravimetric Analysis

**Figure 4:**
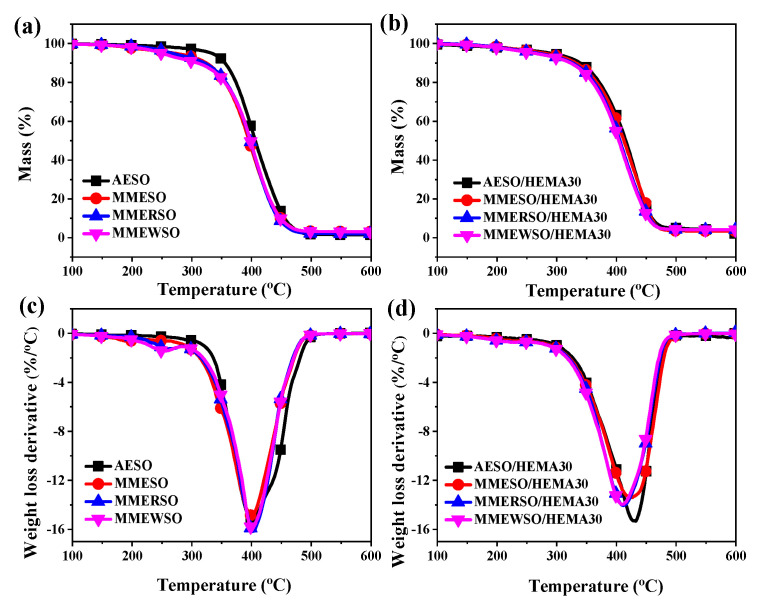
TGA curves (**a**,**b**) and their derivatives (**c**,**d**) of the UV-cured EA-like materials.

TGA analysis of the UV-cured EA-like materials is depicted in Figure 4 and the corresponding data involving 5% weight-loss temperature (*T*_5_), the peak temperature of the weight-loss rate curves (*T*_p_), and char yield (*W*_char_) are summarized in Table 3. Firstly, the *T*_5_ values for the pure EA and EA/HEMA materials were clearly lower than those of the AESO counterparts. This is because the EA prepolymers contained more ester groups than AESO, which is detrimental to the initial thermal stability of the resulting polymeric materials [2,44]. Differently, the *T*_p_ values of the pure EA materials were almost the same as those of the pure AESO material, while the *T*_p_ values of the EA/HEMA materials were lower than those of the AESO/HEMA material. The decline of *T*_p_ probably results from that the EA/HEMA materials showed a larger drop of *ν*_e_ than the AESO/HEMA material when incorporating HEMA into the materials. Secondly, for the pure EA materials, as the C=C functionality increased, the *T*_5_ values increased from 251.0 to 271.1 °C, while the *T*_p_ values fluctuated at about 400 °C. However, for the EA/HEMA materials, both *T*_5_ and *T*_p_ values increased clearly with the increase of C=C functionality. The rise of *T*_5_ and *T*_p_ values is probably ascribed to the growth of *ν*_e_ for both the pure EA and EA/HEMA systems. In addition, the obtained EA/HEMA materials showed better *T*_5_ and *T*_p_ values than the neat EA materials, indicating that the incorporation of HEMA diluent had a positive effect on the thermal stability of the EPO-based materials.

#### 3.4.3. Mechanical Properties

**Figure 5:**
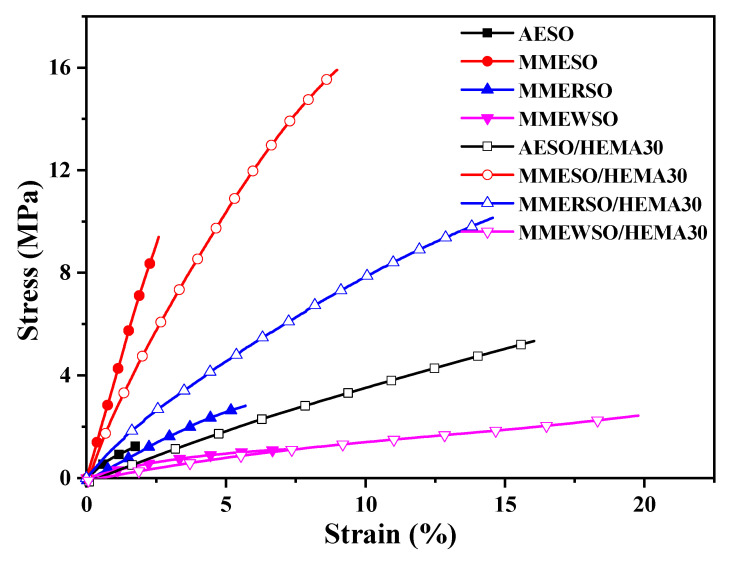
Stress–strain curves of the UV-cured EA-like materials.

Figure 5 exhibited the typical stress–strain curves of the resulting EA-like materials, and the related data are listed in Table 4. Notably, the neat MMESO material exhibited tensile strength and modulus (*σ* and *E*) of 9.42 MPa and 200.3 MPa, respectively, which was 9.2 and 3.4 times the neat AESO resin. The neat MMERSO material also indicated much better tensile *σ* and *E* than the neat AESO material, although ERSO had an obviously lower epoxy value that ESO. Similar results were discovered in the cured EA materials with HEMA diluent. These results also demonstrated that the MAAMA modification of epoxidized plant oils was much more effective than the AA modification for high-performance oil-based epoxy acrylate resins, which agreed very well with the DMA results mentioned above. For the pure EA materials, as the C=C functionality increased, *σ* improved from 1.17 to 9.42 MPa, *E* improved from 19.4 to 200.3 MPa, and breaking strain (*ε*) decreased from 6.26% to 2.49%, respectively. Similar trends were demonstrated for the EA/HEMA materials. These variations can be attributed to the increase of cross-link density. As we know, an increase in cross-link density usually results in the improvement of stiffness and decrease of flexibility and toughness, and vice versa [30,51]. In addition, as the HEMA diluent content increased from 0 to 30%, the values of *σ*, *E,* and *ε* improved remarkably. For instance, the cured MMESO/HEMA30 material exhibited a *σ* value of 15.81 MPa and *E* value of 259.2 MPa, which were comparable to the similar ESO-based UV-curable coatings [7]. The obtained MMERSO/HEMA30 possessed a *σ* value of 10.12 MPa, *E* value of 186.7 MPa, and *ε* value of 14.56, which were 3.5, 2.4, and 2.6 times the values of the pure MMERSO material, respectively. These results meant that the addition of HEMA diluent not only enhanced the rigidity of the resin but also improved the flexibility and toughness of resins. The reason for these changes lies in that the incorporation of HEMA could introduce methyl steric hindrance in the resulting bio-based materials, which was in good accordance with the DMA results, too.

#### 3.4.4. Coating Properties

The coating properties of the cured EA-like materials were analyzed, and the related results are listed in Table 4. Firstly, the pure MMESO film exhibited better adhesion and pencil hardness than the neat AESO film, which is most likely because of the higher *ν*_e_. The neat MMERSO and MMEWSO films possessed equal adhesion, pencil hardness, and flexibility as those of neat AESO film. However, compared to the AESO/HEMA30 materials, the UV-cured EA/HEMA materials demonstrated lower pencil hardness, which is possibly ascribed to the larger drop of *ν*_e_. Secondly, as the C=C functionality rises, the adhesion and pencil hardness of the neat EA-like films increased slightly, while only the pencil hardness of the EA/HEMA films increased apparently. The increase in hardness can also be attributed to the improvement of *ν*_e_. Finally, a significant improvement of adhesion and pencil hardness values was observed when incorporating 30% of HEMA diluent, indicating that the addition of HEMA diluent had a positive impact on the coating performances.

### 3.5. UV-Curing Kinetics of the EA-Like Resins

**Figure 6:**
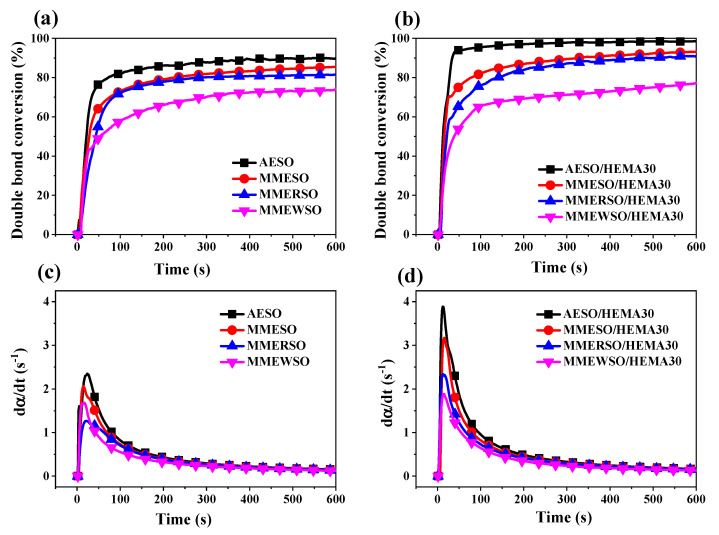
Double bonds conversion rates (**a**,**b**) and polymerization rates (**c**,**d**) of the UV-cured EA-like materials.

Photopolymerization behaviors of the EA-like resins were measured by the Real-time Fourier Transform Infrared Spectroscopy (RT-IR) technique. The real-time C=C conversion of liquid resin was analyzed by monitoring the 810 cm^−1^ peak intensity [43,44], as shown in Figure 6. Obviously, the C=C functional groups of both EA and EA/HEMA materials were rapidly polymerized and reached the highest rates of C=C conversion (d*α*/d*t*) in less than 50 s, and the C=C conversions were all above 60% after 150 s. Therefore, we can conclude that they have excellent copolymerization reactivity. As the C=C functionality increases, the final C=C conversion of the pure EA materials improved from 74.7% to 85.0%. For the EA/HEMA materials, the final C=C conversion improved from 77.1% to 92.9%. When incorporating 30% of HEMA diluent into the EA prepolymers, the final C=C conversions were also improved, which is probably caused by the good diluting effect of HEMA [21,22]. Specially, the pure MMESO resin demonstrated a lower final C=C conversion compared to the AESO resins, which is mainly caused by the effect of a steric hindrance for both maleic and allylic C=C groups in the oil-based EA systems. However, due to the higher C=C functionality of the MMESO oligomer compared to that of the AESO, the resulting MMESO material still indicated higher cross-link density when cross-linking [30]. Similar results were demonstrated when comparing the MMESO/HEMA30 materials with the AESO/HEMA30 materials.

## 4. Conclusions

In this study, we firstly synthesized a series of novel oil-based EA-like prepolymers with different C=C functionality through the modification of epoxidized plant oils with MAAMA, which is a new unsaturated carboxyl acid compound with two active C=C groups and a methyl group. Both the UV-cured pure MMESO and MMESO/HEMA materials showed much better stiffness and heat resistance such as *E′*_25_ *T*_g_, *σ*, and *E* than the corresponding AESO materials. Both the UV-cured pure MMERSO and MMERSO/HEMA materials also exhibited clearly better stiffness and heat resistance than the corresponding AESO materials, although ERSO had an obvious lower epoxy value that ESO. These results indicated that the MAAMA modification of epoxidized plant oils was much more effective than the AA modification for preparing high-performance oil-based epoxy acrylate resins. In addition, we investigated two important effects, the C=C functionality of such EA-like prepolymers and the incorporation of HEMA diluent, on the ultimate properties and UV-curing behaviors of the resulting UV-cured materials and established the structure–property relationships successfully for the obtained EA-like materials. By the increase of C=C functionality of the EA prepolymers or the incorporation of HEMA diluent, most of the important properties including *E’*_25_, *T*_g_, *T*_5_, *T*_p_, *σ*, *E*, and hardness of coating increased generally for both the pure EA and EA/HEMA resins. The growths mainly result from the rise of *ν*_e_ or the incorporation of methyl steric hindrance or both. In general, this study could not only provide several high-performance UV-curable oil-based EA resins which can be applied in the fields of coatings such as wood coatings but also offer substantial fundamental research for tuning their properties and the UV-curing process for the coating application.

## Figures and Tables

**Table 1:** Composition of UV-cured epoxy acrylate (EA)-like samples. HEMA: hydroxyethyl methacrylate.

Samples *	EA (wt %)	HEMA (wt %)	Darocur 1173 (wt %)
MMESO	100	0	1.5
MMERSO	100	0	1.5
MMEWSO	100	0	1.5
MMESO/HEMA30	70	30	1.5
MMERSO/HEMA30	70	30	1.5
MMEWSO/HEMA30	70	30	1.5

* The epoxy values of ESO, ERSO, and EWSO used for the synthesis of EA-like prepolymers were 7.34%, 5.15%, and 4.38%, respectively.

**Table 2:** Properties of UV-cured EA-like samples. AESO: acrylated ESO.

Samples	*A*_v_*^a^* mgKOH/g	*N*_C=C_ *^b^*	*C*_bio_^*c*^ (%)	*C*_gel_^*d*^ (%)
AESO	18.2	2.25	84.1	93.1
MMESO	20.2	7.81	59.0	95.9
MMERSO	23.2	5.92	65.1	93.3
MMEWSO	22.4	4.40	71.3	87.1
AESO/HEMA30	-	-	58.8	98.0
MMESO/HEMA30	-	-	41.3	99.9
MMERSO/HEMA30	-	-	45.6	99.5
MMEWSO/HEMA30	-	-	50.0	98.5

*^a^* Acid value. *^b^* Introduced C=C functionality per triglyceride. *^c^* Bio-based. content. *^d^* Gel content.

**Table 3:** Dynamic Mechanical Analysis (DMA) and Thermogravimetric Analysis (TGA) results of the UV-cured EA-like materials.

Samples	*E’*_25_*^a^* (MPa)	*T*_g_*^b^* (°C)	*E’*_Tg + 60_*^c^* (MPa)	*ν*_e_*^d^* (×10^3^ mol/m^3^)	*T*_5_*^e^* (°C )	*T*_p_*^f^* (°C)	*W*_char_*^g^* (%)
AESO	99.2	15.7	31.6	3.63	331.2	401.0	1.54
MMESO	697.5	69.4	57.6	5.74	271.1	399.8	3.01
MMERSO	343.5	55.6	28.8	2.97	265.9	401.6	1.96
MMEWSO	42.6	17.2	7.5	0.86	251.0	399.8	3.08
AESO/HEMA30	550.9	56.2	18.6	1.91	292.2	432.2	2.14
MMESO/HEMA30	1017.2	84.0	19.8	1.92	281.3	426.3	3.37
MMERSO/HEMA30	695.7	73.9	12.2	1.20	271.4	411.3	4.09
MMEWSO/HEMA30	43.7	17.5, 107.8	2.7	0.25	263.8	408.9	4.05

*^a^* Storage modulus at 25 °C. *^b^* Glass transition temperature, for MMEWSO/HEMA30 system, the second ones were employed to calculate cross-link density. *^c^* Storage modulus at *T*_g_ + 60 °C. *^d^* Cross-link density. *^e^* 5% Thermal weight loss temperature. *^f^* Peak temperature at the curves of the weight-loss rate. *^g^* Char yield.

**Table 4:** Mechanical properties and coating performance of the UV-cured EA-like materials.

Samples	*σ ^a^* (MPa)	*E ^b^* (MPa)	*ε ^c^* (%)	Ad*. ^d^* (Grade)	P.H. ^e^	Fl *^f^* (mm)
AESO	1.02 ± 0.17	59.5 ± 8.2	1.75 ± 0.91	4	6B	2
MMESO	9.42 ± 0.79	200.3 ± 29.0	2.49 ± 0.86	3	5B	2
MMERSO	2.90 ± 0.32	76.6 ± 5.4	5.55 ± 0.79	4	6B	2
MMEWSO	1.17 ± 0.26	19.4 ± 2.4	6.26 ± 1.33	4	6B	2
AESO/HEMA30	5.30 ± 0.12	83.3 ± 5.0	15.61 ± 0.56	2	2H	2
MMESO/HEMA30	15.81 ± 0.13	259.2 ± 7.3	8.99 ± 0.60	3	H	2
MMERSO/HEMA30	10.12 ± 0.55	186.7 ± 6.9	14.56 ± 1.11	2	2B	2
MMEWSO/HEMA30	2.41 ± 0.29	11.8 ± 0.6	19.58 ± 1.36	2	4B	2

*^a^* Tensile strength. *^b^* Tensile modulus. ^*c*^Elongation at the break. *^d^* Adhesion. *^e^* Pencil hardness. *^f^* Flexibility.

## Supplementary Materials

The following are available online at https://www.mdpi.com/2073-4360/12/9/2165/s1, Figure S1: Acid values of MAAMA, Figure S2: FT-IR spectra of (a) ERSO, (b) MMERSO, (c) EWSO, and (d) MMEWSO, Figure S3: ^1^H NMR spectrum of (a) ERSO, (b) MMERSO, (c) EWSO, and (d) MMEWSO, Figure S4: ^1^H NMR spectrum of MAAMA, Figure S5: ^1^H NMR spectrum of ESO, Figure S6: ^1^H NMR spectrum of ERSO, Figure S7: ^1^H NMR spectrum of EWSO, Figure S8: ^1^H NMR spectrum of MMESO, Figure S9: ^1^H NMR spectrum of MMERSO, Figure S10: ^1^H NMR spectrum of MMEWSO, Figure S11: ^1^H NMR spectrum of AESO, Equation (S1): Determining the grafted C=C functionality for AESO.
