# Supplementary material for: Preparation and Properties of Plant-Oil-Based Epoxy Acrylate-Like Resins for UV-Curable Coatings
Source: Polymers (Basel). 2020 Sep 22;12(9):2165. doi: 10.3390/polym12092165 (PMC7570069; doi:10.3390/polym12092165)
Supplement: Supplementary file 1 [file polymers-12-02165-s001.pdf]

Supporting Information

Preparation and Properties of Plant-Oil-Based Epoxy Acrylate-like Resins for UV-Curable Coatings

Jijun Tang [1,†], Jinshuai Zhang [2,†], Jianyu Lu [1,2], Jia Huang [2], Fei Zhang [2], Yun Hu [2], Chengguo Liu [2,*], Rongrong An [3,*], Hongcheng Miao [2,4], Yuanyuan Chen [2,4], Tian Huang [2,4] and Yonghong Zhou [2,*]

Figures. S1–S11

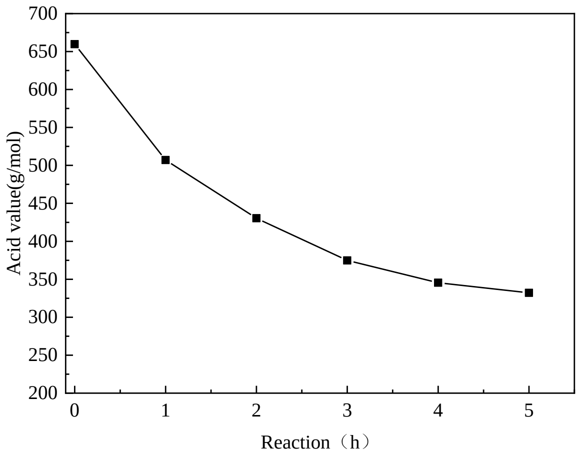

Figure. S1 Acid values of MAAMA

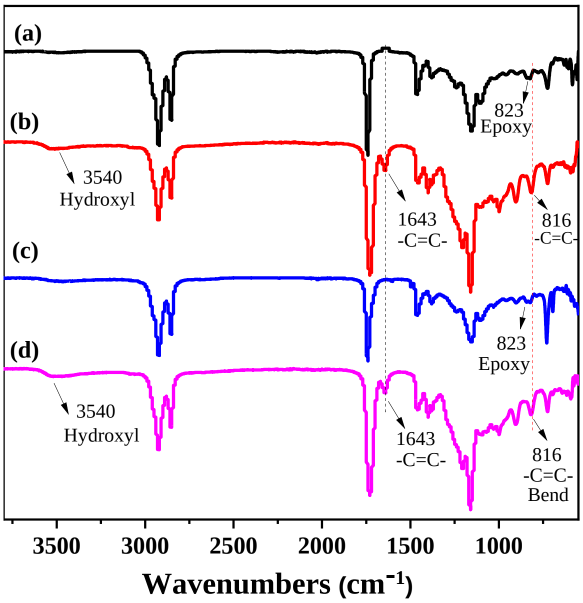

Figure. S2 FT-IR spectra of (a) ERSO, (b) MMERSO, (c) EWSO, and (d) MMEWSO

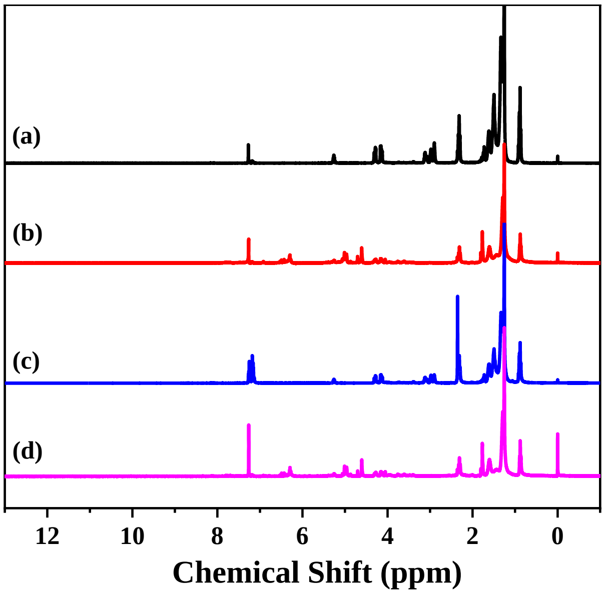

Figure. S3 ¹H NMR spectrum of (a) ERSO, (b) MMERSO, (c) EWSO, and (d) MMEWSO

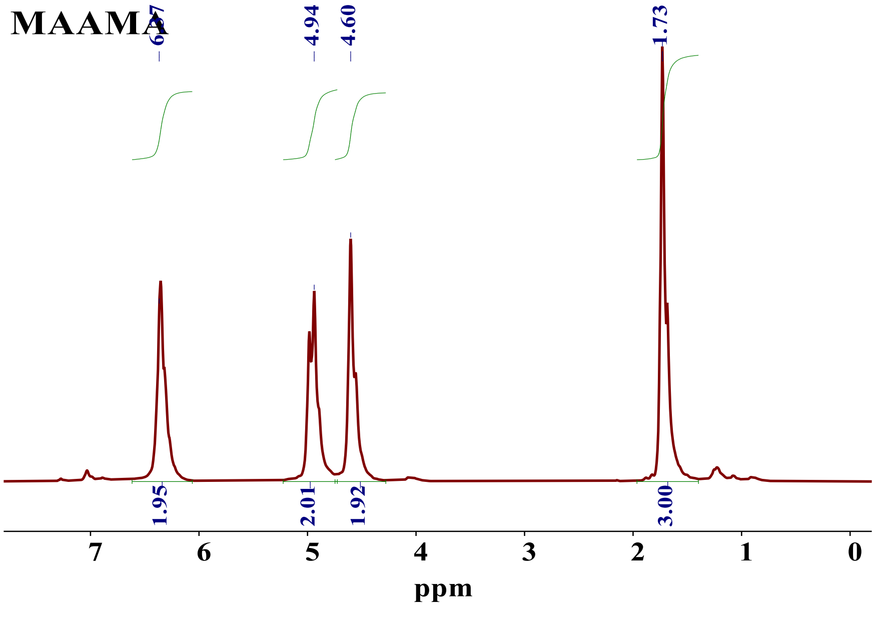

Figure. S4 ¹H NMR spectrum of MAAMA

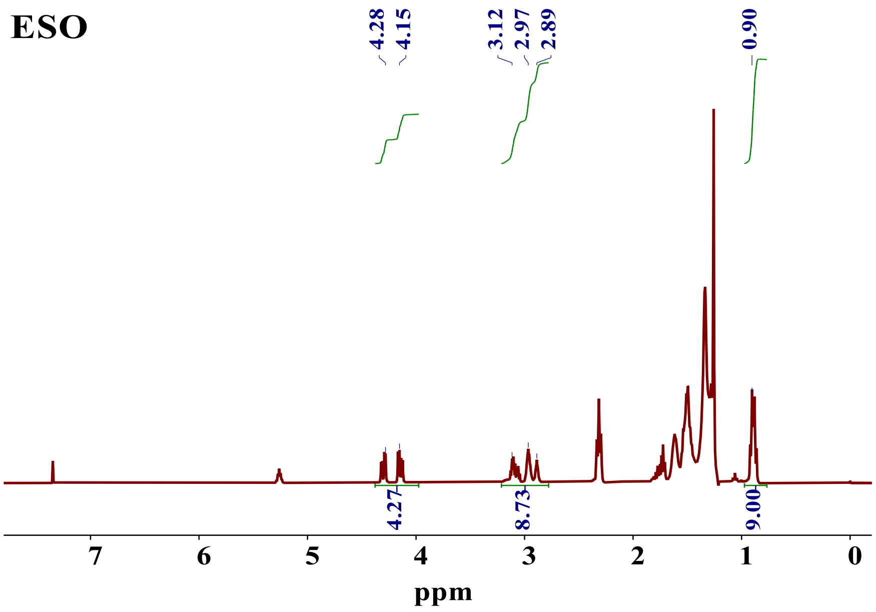

Figure. S5 ¹H NMR spectrum of ESO

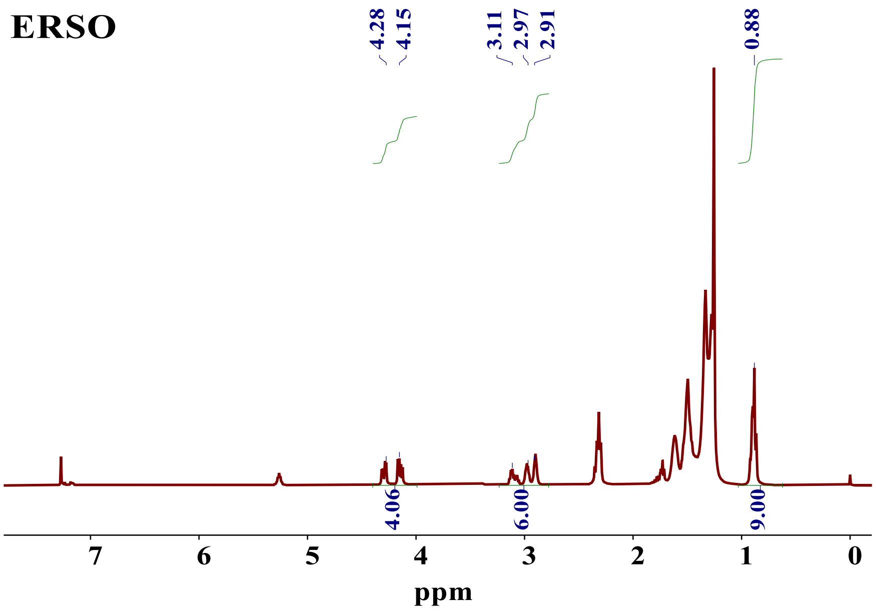

Figure. S6 ¹H NMR spectrum of ERSO

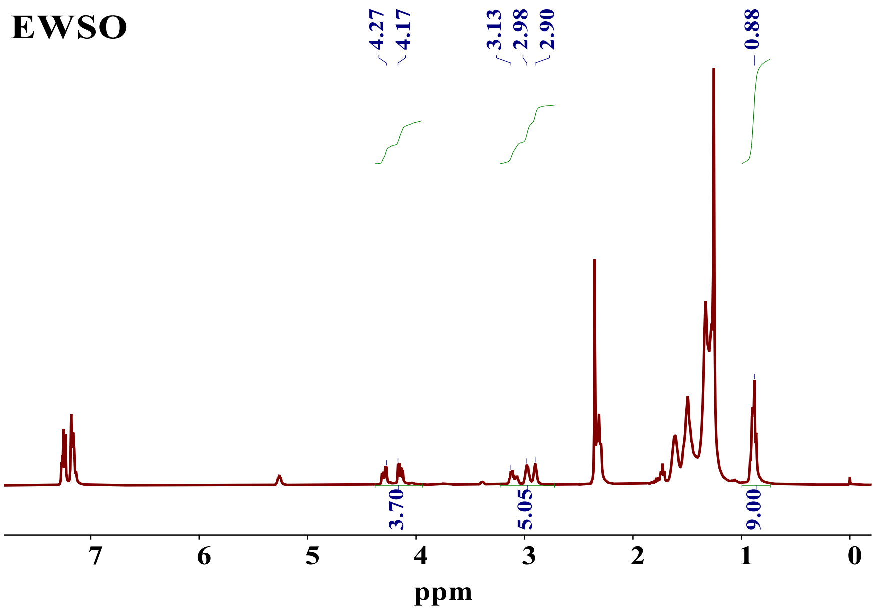

Figure. S7 ^{1}H NMR spectrum of EWSO

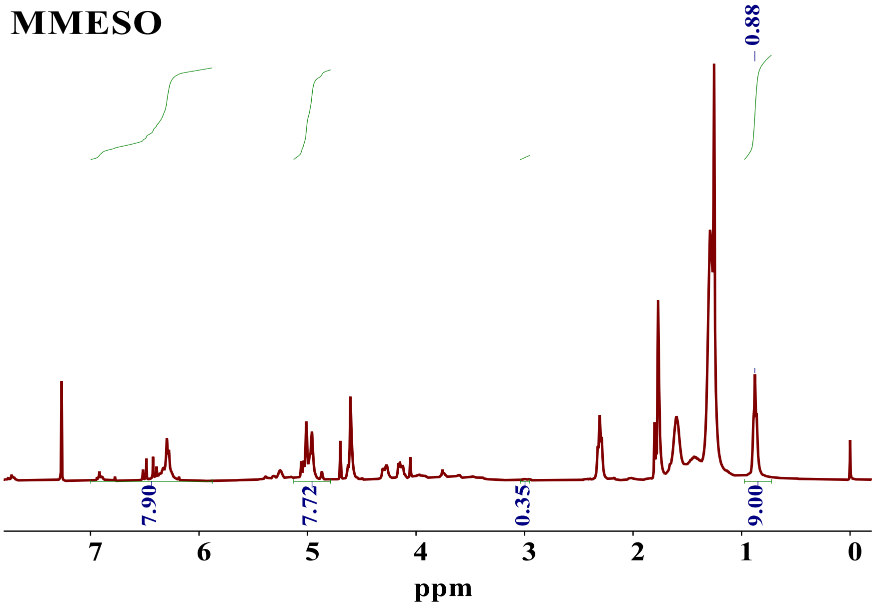

Figure. S8 ^{1}H NMR spectrum of MMESO

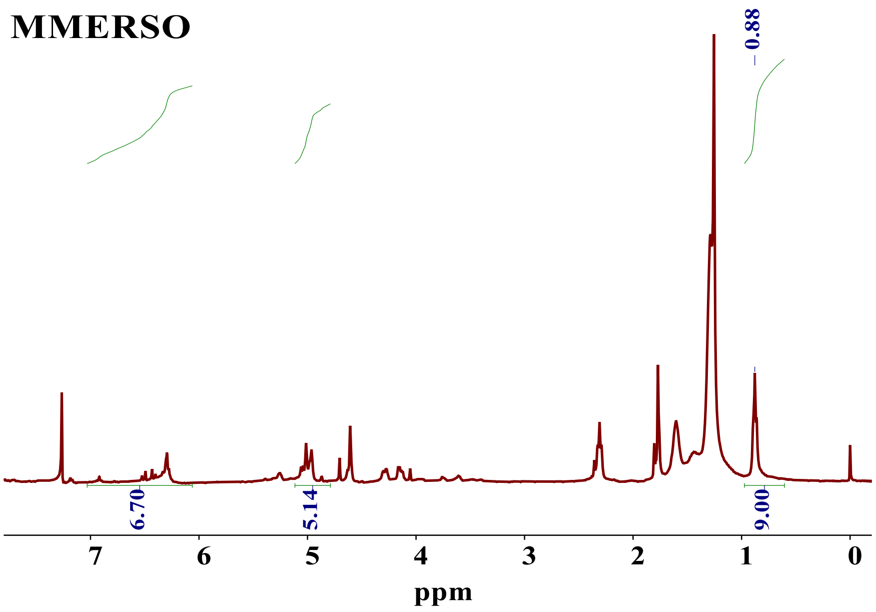

Figure. S9 ^{1}H NMR spectrum of MMERSO

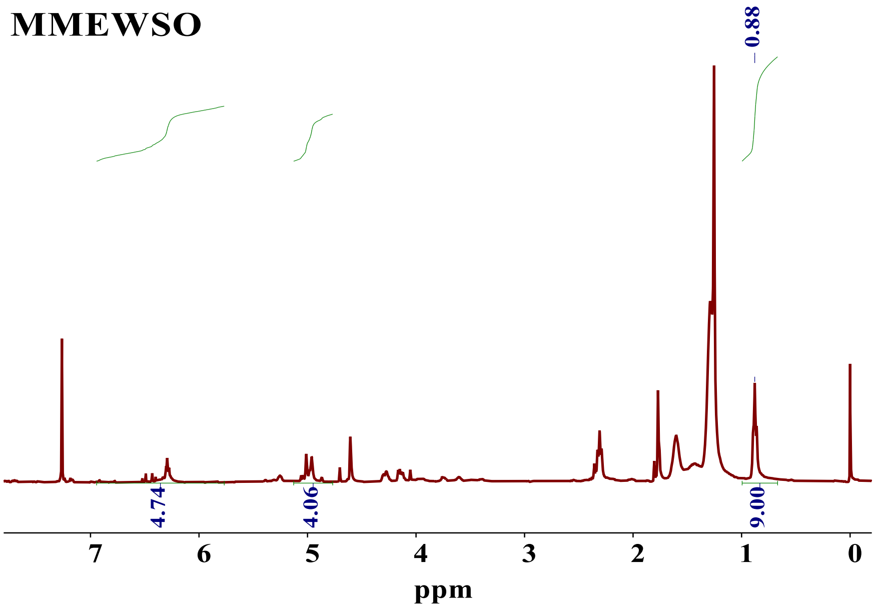

Figure. S10 ^{1}H NMR spectrum of MMEWSO

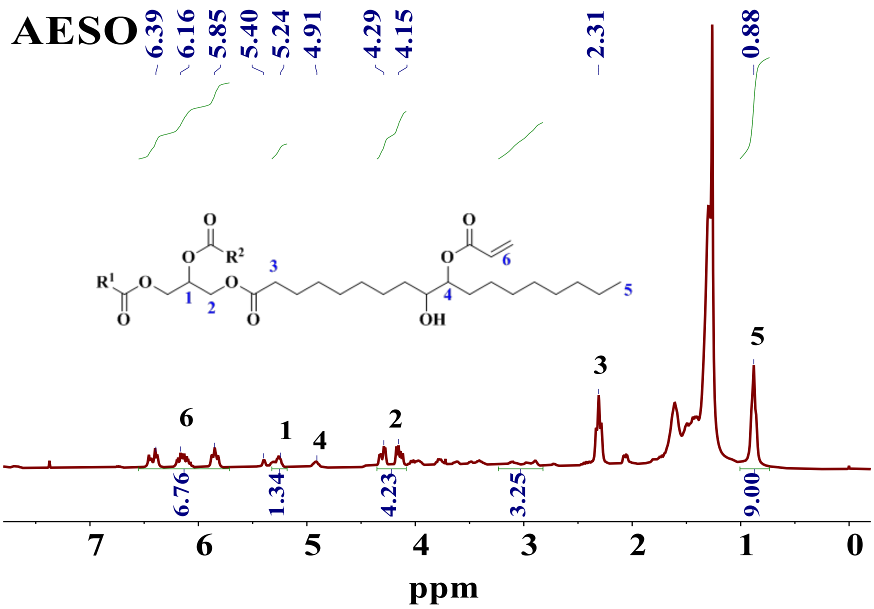

Figure. S11 ^{1}H NMR spectrum of AESO

Equation. S1 Determining the grafted C=C functionality for AESO

$$N_{C=C} = \frac{A_{5.6\text{-}6.7\,\mathrm{ppm}}/3}{A_{0.88\,\mathrm{ppm}}/9} = \frac{3A_{5.6\text{-}6.7\,\mathrm{ppm}}}{A_{0.88\,\mathrm{ppm}}} \qquad \textbf{(S1)}$$
